# Positive regulation of root-knot nematode control in *Capsicum* through bioactive compounds derived from plant seeds cake

**DOI:** 10.1038/s41598-025-06467-y

**Published:** 2025-06-27

**Authors:** Mostafa Sayed Mostafa El-Ansary, Waleid Kottb Hegazy, Ragaa A. Hamouda

**Affiliations:** 1 Department of Plant Biotechnology, Genetic Engineering and Biotechnology Research Institute (GEBRI), University of Sadat City, Sadat City, Egypt; 2 Microbial Biotechnology Department, Genetic Engineering and Biotechnology Research Institute, University of Sadat City, Sadat City, Egypt; 3 Department of Applied Radiologic Technology, College of Applied Medical Sciences, University of Jeddah, Jeddah, 23218 Saudi Arabia

**Keywords:** *Meloidogyne incognita*, Plant control, Pepper plant, Seeds cake, Bio-active compounds, Biotechnology, Plant sciences

## Abstract

Plant-parasitic nematodes (PPNs) pose a significant problem for farmers worldwide, leading to yield losses. Several conventional strategies, such as artificial nematocides, have been used in the past to control PPNs in pepper plants. In an in vivo trial aimed at reducing root-knot nematodes, (RKNs) *Meloidogyne incognita* communities in soil and root infestation, certain plant seed cake (PSC) was evaluated for its potential use. In this study, four PSCs were used to manage PPNs: black seed, jojoba, olive, and jatropha. These PSCs relatively inhibited nematode reproduction and promoted pepper plant health. Notably, black seed and jojoba were the most effective toxic PSC against RKNs, *M. incognita*, especially targeting the second-stage J2s in soil. For example, treatment with black seed at both 15 and 30 g rates, as well as jojoba at 15 g rate, was consistently effective in reducing the final nematode population. Growth parameters, including shoot and root weight and length, as well as the number of leaves, were measured. The results showed that black seed at 30 g and jojoba at 15 g significantly increased shoot weight, followed by black seed at 15 g, with corresponding values of 75.89 g, 47.86 g, and 45.9 g, respectively. According to GC-MS analysis, the mode of action of these PSC may involve natural active compounds capable of killing or inhibiting nematode communities. The GC-MS analysis of jatropha seeds cake showed remarkable bioactive compounds, including D-Psicofuranose, pentakis (trimethylsilyl) ether (isomer 2); 9,12-Octadecadienoic acid; 2-((2-Methyl-1-oxa-4-azaspiro [4.4]non-4yl) carbonyl) cyclopropane carboxylic acid and 1 H-Indene, 2,3-dihydro-4-propyl. These compounds have antimicrobial, insecticidal, anti-nematodal, and antiviral activities confirming their potential as natural biopesticides.

## Introduction

Plant-parasitic nematodes (PPNs), including root-knot nematodes (RKNs), are among the most damaging pests in crop systems, significantly limiting agricultural yield^1^. Most chemical nematocides are highly resistant to biodegradation, leading to their accumulation in the soil and causing severe environmental toxicity^2^. Higher plant species have phytochemical components that are antagonistic to RKNs^6^. These plants exhibit a broad-spectrum mode of action, primarily due to the presence of monoterpenes and sesquiterpene^4^. These two components often have strong repellent effects, particularly when utilized as pest-repelling essential oils^102^. For example, several plant cake extracts of *Nicotiana tabacum*, *Azadirachta indica*, *Syzygium aromaticum*, and *Crotalaria* spp. demonstrated notable nematocidal effects against RKNs^3^.

Research on bio-agents that target RKNs without harming the environment is becoming increasingly important^4^. Low-cost and eco-friendly substitutes are especially beneficial^103^. In addition, secondary metabolites extracted from certain higher plants have a direct impact on various stages of RKNs^5^.

Plants produce thousands of structurally diverse, low-molecular-weight organic compounds^104^. Many of these compounds are toxic to insects, nematodes, and other organisms. These plant-derived compounds contribute significantly to the biological control of soil pests^6^.

Phytochemical analysis showed the presence of various components in many plant cakes, including anthroquinones, alkaloids, amino acids, tannins, saponnins, flavonoids, proteins, terpenoids^7^. *Nigella sativa* seeds are used for flavoring, herbal medicine, and oil production. Furthermore, medicinal purposes phytochemical analysis indicated that they have many important bioactive compounds^8^. Similarly, jatropha seed cake contains several anti-nutritional compounds including saponin, phytate, trypsin inhibitors, glucosinolates, amylase inhibitors, flavonoids, vitexine, isovitexine and cyanogenic glucosides, as well as toxic irritant compounds^9^. Jojoba seed cake contains cyanoglucosides and simmondsins which are highly toxic to rats, mice, chickens, and sheep^10^. Olive cake, (*Olea europaea*, family Oleaceae), contains hetero cyclic compounds with antimicrobial activity against some Gram-positive and Gram-negative bacterial species^11^. Olive cake is considered a cheap and readily available source of phenolic compounds^12^.

The mode of actions of bioactive compounds against nematode reported as the following, the terpenoids and phenolics repel nematodes or inhibit their ability to locate hosts. Glycoside compounds release cyanide upon hydrolysis which is toxic to nematodes. Some alkaloids immobilized nematodes. Compounds like allyl isothiocyanate and asparagusic acid inhibit nematode egg hatching. Some compounds, such as betaines, suppress nematode reproduction indirectly by altering host plant physiology^4^.

The current research aims to evaluate the bioactive compounds of PSC, such as *N. Sativa*; *S. chinesis*; *O. europaea*, and *J. curcas* against RKNs, *M. incognita* infecting pepper. Using the GC-MS technique, this study may provide insights into the effectiveness of using natural nematocides as alternatives to toxic chemicals.

## Results and discussion

### Nematicidal screening trial

#### The effect of botanical seed extracts on *M. incognita*

**Fig. 1:**
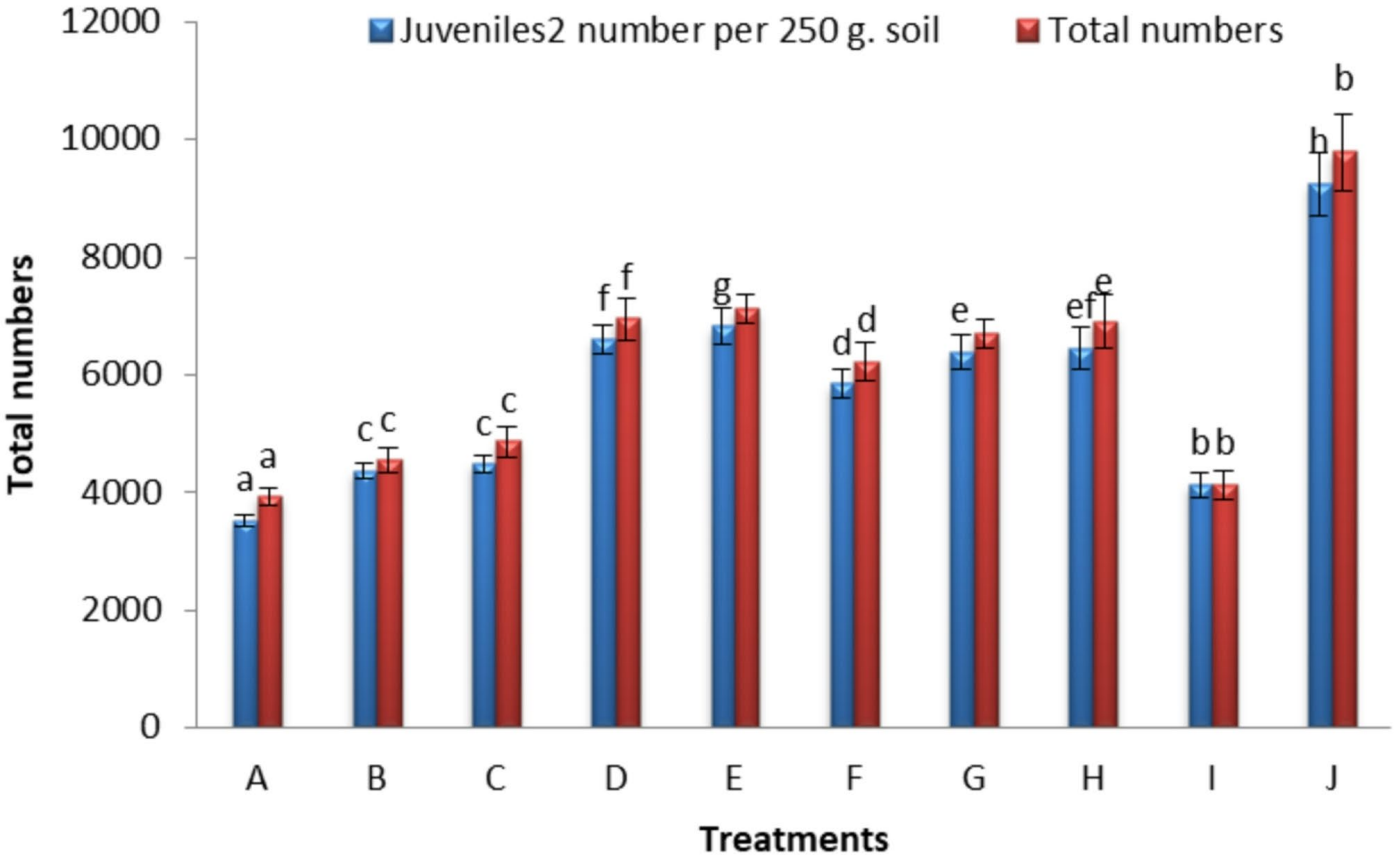
Effect of some plants’ cake extracts on *M. incognita* J2s numbers in soil and total numbers per plant.

**Fig. 2:**
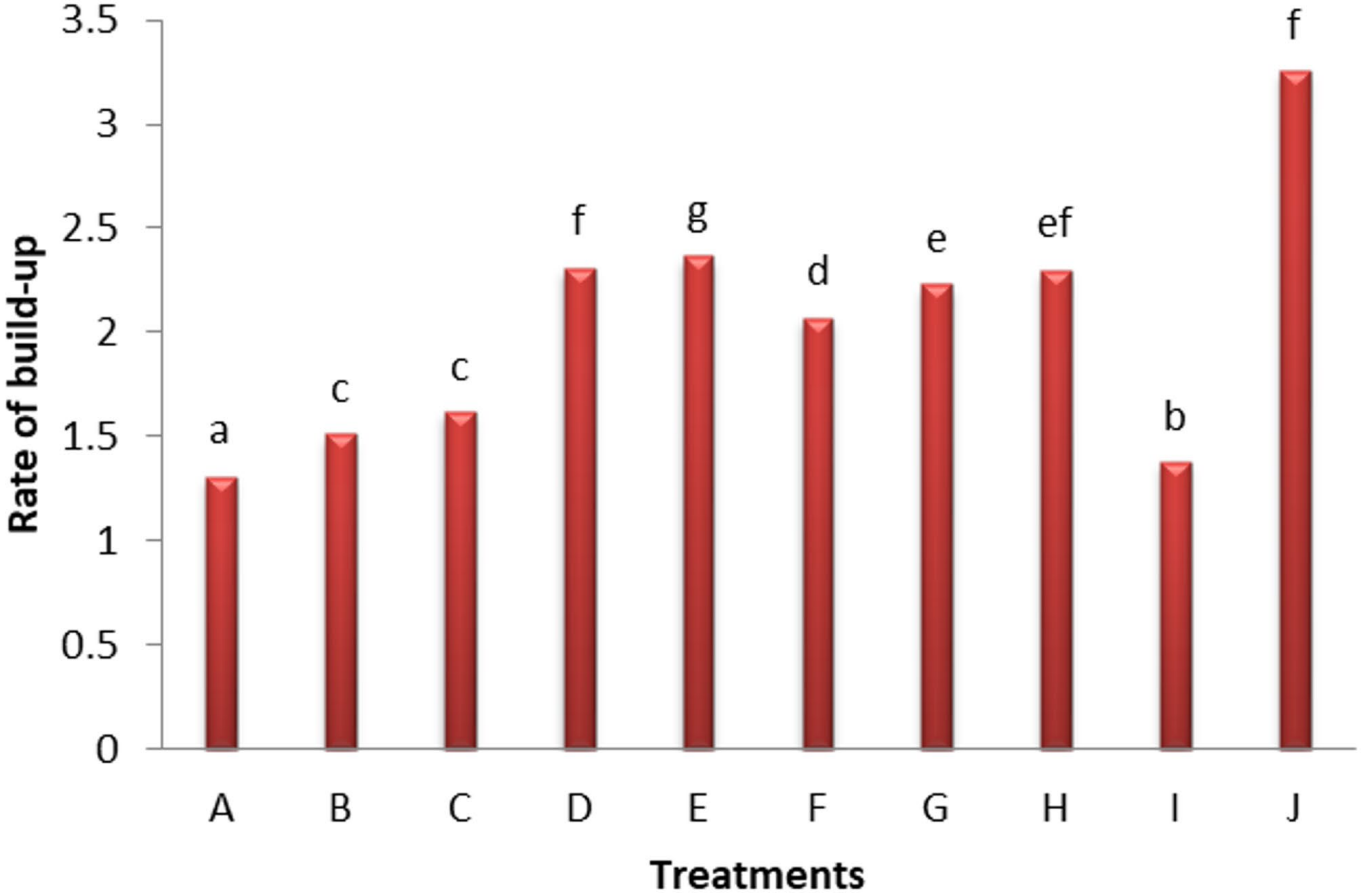
Evaluation of some plant cake extracts on the rate of nematode build-up in in vivo.

In an in vivo trial, we aimed to reduce the RKN, *M. incognita* populations in the soil and minimize pepper plant infestation using natural materials (Table 1). The application of jatropha, neem, and castor oil cakes significantly enhanced plant growth and reduced *M. incognita* infestation compared to the control. The water-soluble fractions of jatropha cake, at 10% concentration, increased J2s mortality to 49.33% after 48 h of incubation^15^. In recent years, plant extracts, such as seed oil cake have been widely used for managing PPNs. Notably, oil seed cakes extract of some plants such as *Niglla sativa*, *Simmondsia chinesis*, *Olea europaea*, and *Jatroph curcas* have demonstrated a relative inhibition of nematode reproduction and promotion of plant health^16^. Also, *N. Sativa* and *S. chinesis* were the most effective toxic cakes against RKN, *M. incognita*, specifically targeting second-stage juveniles (J2s) in the soil (Fig. 1). However, plant cake extracts of *Nicotiana tabacum*, *Azadirachta indica*, *Syzygium aromaticum*, and *Crotalaria* spp. have mainly bio-nematocidal effect on RKNs^3^. Additionally, treatment with black seeds cake (A&B) at both rate 15 and 30 g, as well as (C) jojoba at a rate of 15 g, significantly reduced the number of galls (recorded at 188, 107.9 and 179.5), as well as J3, J4, females, egg masses, and J2s in soil and total nematode count and the build-up rate (Fig. 2). In general, it can be concluded that black seed cakes (A & B) were the most effective material in reducing RKN (*M. incognita*) build-up, followed by jojoba (C) with reduction rates of 1.31, 1.51, and 1.62 times, respectively.

**Table 1:** Reproduction and nematode build-up of *M. incognita* in pepper roots (Dolma 292).

Treatments*	Galls	J 3	J 4	Females		Egg-masses	Juveniles2 number per 250 g. soil	Total numbers**	Rate of build-up***
	Number/5 g. root								
A	188 a	36.1 a	48.7 b		148.1 de	179.1 ab	3524 a	3937 ab	1.31 ab
B	107.9 bc	29.5 c	29.5 a		96.5 bcd	72.0 a	4370 a	4538.5 a	1.51 a
C	179.5 ab	57.4 ab	52.3 b		103.3 bcd	175.0 ab	4486 a	4874 ab	1.62 ab
D	187.4 bc	46.3 c	39.4 b		87.1 bc	166.6 ab	6610 b	6949.4 b	2.31 b
E	184.5 c	31.5 c	35.0 b		53.5 ab	173.7 ab	6828 b	7121.7 b	2.37 b
F	207.5 bc	30.5 bc	33.0 b		114.5 cd	199.5 b	5846 ab	6223.5 ab	2.07 ab
G	205.9 bc	30.5 c	32.5ab		99.0 bcd	155.5 ab	6384 ab	6701.5 b	2.23 b
H	231.5 bc	29.5 c	40.0 b		172.5 e	210.0 bc	6452 b	6904 b	2.3 b
I	190.0 bc	31.2 c	33.5 a		32.0 a	76.5 a	3792 b	4139.7 a	1.38 ab
J	362.5 d	33.5 d	53 c		87.0 bc	363.0 c	9254 c	9790.5 c	3.26 c

*Treatments: (A) *N. sativa* 15 g, (B) *N. sativa* 30 g, (C) *S. chinensis* 15 g, (D) *S. chinensis* 30 g, (E) *O. europaea* 15 g, (F) *O. europaea* 30 g, (G) *J. curcas* 15 g, (H) *J. curcas* 30 g, I), Nematicides check, Oxamyl 0.5 ml (Vydate 24% L {Vydate L24%, Methyl-N, N-dimethyl N- (Methyl carbamayl) oxythioxamidate}); J), Check with infection; K), Check without infection.

Means followed by the same letter (s) within a column are not significantly different (*p* ≤ 0.05) according to Duncan’s multiple range test.

**Total population including = developmental stages (J3&J4) + females + egg-masses + juveniles number in soil (J2s).

***Rate of nematode build-up = pf (final population/initial population).

Finally, the oil seed cakes of the tested plants in this study demonstrated a predominantly antagonistic effect against RKN. However, their mode of action may be attributed to natural nematotoxic compounds that can either kill or inhibit nematode populations. Some of such materials contain various natural chemical compounds, e.g., alkaloids, phenols, terpeniods, organic acids, glycosides, limonoids, quassinoids, and volatile oils^1^&^17^.

### Plant health

The effectiveness of the applied treatments in inhibiting the reproduction of *M. incognita* had a positive impact on the growth and health of pepper plants. Growth parameters, including weights and lengths of shoots and roots as well as the number of leaves, were measured (Table 2). For example, treatment with black seed (B) at 30 g. and jojoba (C) at 15 g resulted in a significant increase in shoot weight, followed by black seed (A) at 15 g. (75.89, 47.86 and 45.9 g., respectively). On the contrary, no remarkable increase in root length was observed. However, the application of jatropha, neem, and castor oil cakes considerably improved plant health and lessened host infestation by RKN, *M. incognita* as well as in the control treatment^15^.

**Table 2:** Effect of PSC treatments on plant health infected with RKN, *M. incognita* in in vivo.

Treatments*	Shoot weight (g)	Shoot length (cm)	Root weight (g)	Root length (cm)	Leaves Number (no.)
A	45.9 de	60.8 bc	33.92 bc	39.2 ab	46.4 cd
B	75.89 e	74.4 c	41.9 c	35.4 b	60.6 e
C	47.86 cd	63.4 bc	28.66 b	32.6 ab	44 cd
D	41.78 cd	55.8 b	31.8 bc	36.2 ab	42.2 bcd
E	23.39 ab	48 a	12.33 a	29.6 a	25 ab
F	27.79 ab	56.4 b	12.77 a	28 ab	25.4 abc
G	43.98 bcd	63.2 bc	28.0 b	33 ab	40.2 bcd
H	53.9 de	71.4 c	24.53 b	34 b	50.8 d
I	17.38 abc	46.2 a	9.62 a	30.4 a	28.6 a
J	14.25 a	47.6 a	13.41 a	27.8 a	17.8 a
K	34.12 bc	65.2 bc	21.13 b	34 b	29.2 a

*Treatments: (A) *N. sativa* 15 g, (B) *N. sativa* 30 g, (C) *S. chinensis* 15 g, (D) *S. chinensis* 30 g, (E) *O. europaea* 15 g, (F) *O. europaea* 30 g, (G) *J. curcas* 15 g, (H) *J. curcas* 30 g, I), Nematicides check, Oxamyl 0.5 ml (Vydate 24% L {Vydate L24%, Methyl-N, N-dimethyl N- (Methyl carbamayl) oxythioxamidate}); J), Check with infection; K), Check without infection.

Means followed by the same letter (s) within a column are not significantly different (*p* ≤ 0.05) according to Duncan’s multiple range test.

### Inter-correlation among all data of infected pepper plants treated with various plant seed cakes (PSCs)

#### Multivariate analysis

**Fig. 3:**
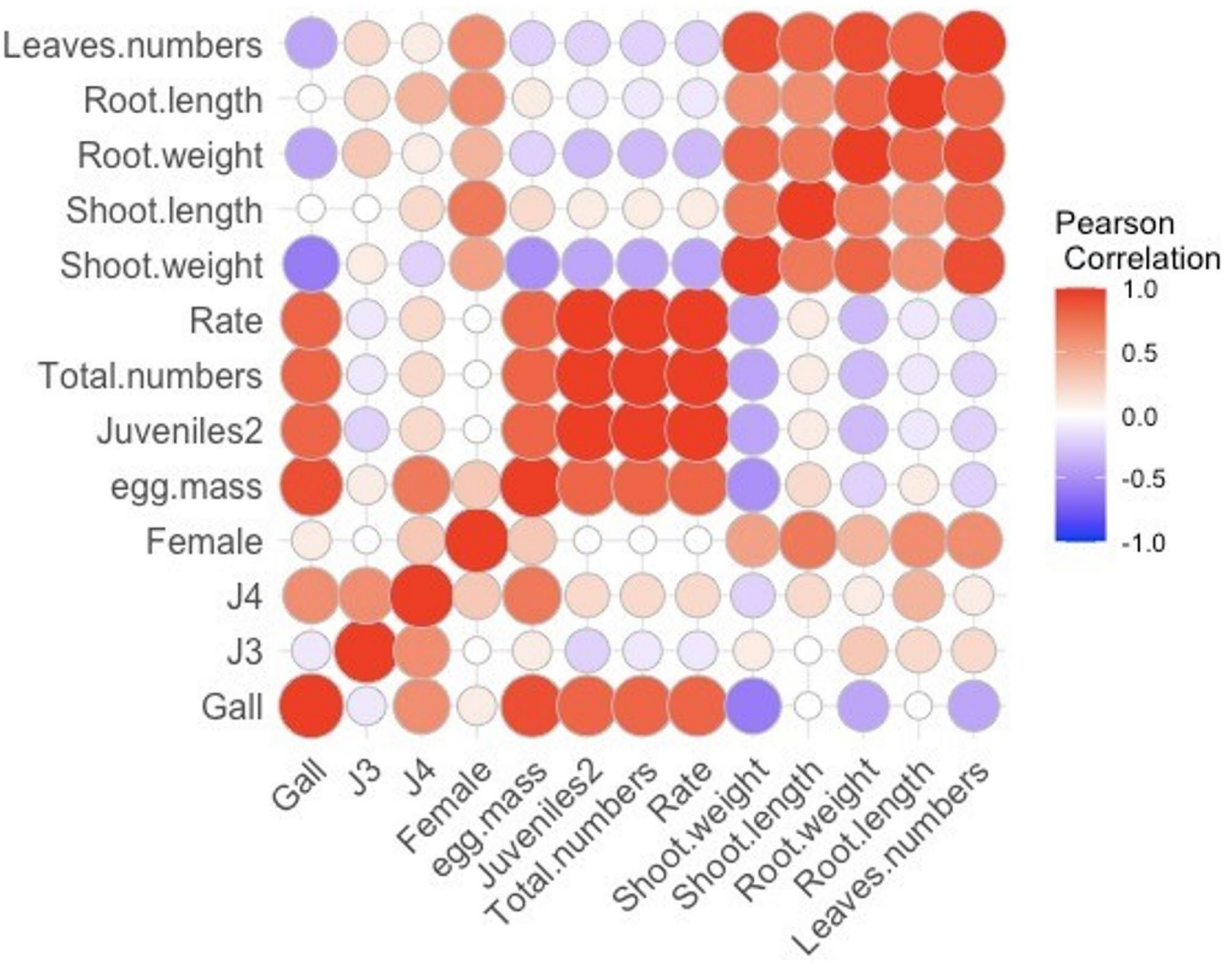
Heat maps and Pearson correlation among all measured data (Reproduction, Rate of build-up, and Plant growth parameters) infecting pepper plants after treatments by different PSCs.

Since multivariate analysis techniques produce more accurate, realistic, and relevant results, they can be used to explore relationships, categorize data, and estimate components or parameters within complex data sets^15^. Figure 3 illustrates the correlation among all measured parameters including, reproduction, and nematode build-up and growth parameters of pepper roots after treatment with different PSCs. The red color indicates a positive correlation among the data, while the blue color highlights a negative one. Figure 3 displays heat maps and Pearson correlation analysis of all measured parameters (reproduction, nematode build-up and growth parameters) of *M. incognita* in infected pepper plants after treatments with PSCs. The blue color represents a negative correlation, while the red color signifies a positive correlation. A strong correlation was observed between galls and egg mass (r^2^ = 0.9279), J3 and J4 with r^2^ (0.7502), juveniles with total numbers r^2^ (0.9976), and juveniles with the rate of build-up (r^2=^ 0.9975). A strong negative correlation was detected among each of the following egg mass, juveniles2, total numbers, and rate of build-up with all growth parameters such as shoot weight, and length, root length, and weight, as well as leaves numbers.

#### Principal component analysis biplot

**Fig. 4:**
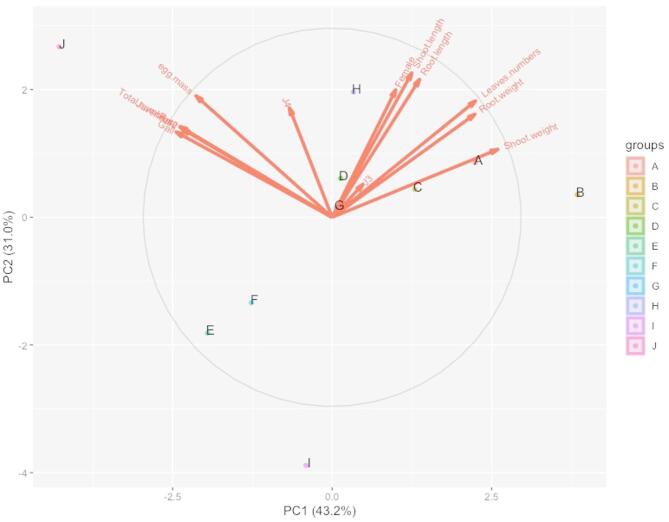
Principal component analysis performed on the dataset of RKNs infecting pepper plants after treatments with different plant seed cakes.

Principal component analysis (PCA) is a multivariate statistical technique used to analyze large and complex datasets. Only principal components with eigenvalues greater than one are considered significant. The PCA technique was applied to assess the item variability based on quality attributes, while a similarity hierarchy was used to categorize the items^19^. The PCA-biplot is one of the most effective multivariate techniques for evaluating trait interactions and it is widely used to analyze trait relationships in various agricultural plants^20^. In the PCA scatter plot of infected pepper plants treated with different PCA formulations, the first two principal components (PCs) together explained 74.4% of the total variation, with PC_1_ accounting for 43.2% and PC_2_ for 31.2% (Fig. 4). Interpretation of the PCA biplot was based on vector distances and cosine angles between variables. Traits such as shoot length, root length, shoot weight, root weight, and RKN developmental stages (female, J3, J4) exhibited strong positive correlations, as indicated by the small angles between their vectors. These traits were identified as the most significant in differentiating treatments A, B, and C. Conversely, egg mass, gall formation, rate of build-up, and juvenile J2s counts were the dominant factors distinguishing treatments H, F, D, E, and G. These results suggest that plant growth parameters and the severity of nematode infestation are the key determinants of treatment efficacy. The PCA-biplot effectively visualized these relationships, highlighting its role as a valuable tool for trait evaluation in agricultural research^108^.

Treatments: (A) *N. sativa* 15 g, (B) *N. sativa* 30 g, (C) *S. chinensis* 15 g, (D) *S. chinensis* 30 g, (E) *O. europaea* 15 g, (F) *O. europaea* 30 g, (G) *J. curcas* 15 g, (H) *J. curcas* 30 g, I), Nematicides check, Oxamyl 0.5 ml (Vydate 24% L {Vydate L24%, Methyl-N, N-dimethyl N- (Methyl carbamayl) oxythioxamidate}); J), Check with infection; K), Check without infection.

### GC-MS analysis

The GC-MS analysis of *Nigella sativa* (black seed) cake identified 11 compounds with antimicrobial and insecticidal properties, confirming their potential biopesticide activity (Table 3).

**Table 3:** Major bioactive compounds extracted from black seed cake, *N. sativa*, using GC-MS analysis.

Rt	Compounds	Types	Area %	Activity	Ref.
6.29–6.91	Bis (trimethylsilyl) trifluoroacetamide	Organosilicon	18.6286	Anti-inflammatory, antibacterial, antiulcerative, and antitumor properties	^21^
10.2224	Glycerol	Sugar alcohol	10.12	Used in pharmaceutical industries	^22^
16.0896	Palmitic acid	Saturated fatty acids	10.69	Induces apoptosis in the human leukemic cell, antitumor activity in vivo in mice.	^23^
17.1275	9,12-Octadecadienoic acid (Z, Z)	Unsaturated Fatty acids	13.68	Antisecretory, antispermigenic, antitonsilitic, antitubercular.	^24^
17.3022	Linoelaidic acid, trimethylsilyl ester	Unsaturated Fatty Acids	16.78	Antioxidant, anti-inflammatory with anticancer agent	^25^
17.3922	Stearic acid	Saturated fatty acid	2.85	Used as the plasticizer in corn zein protein	^26^
18.2501	11,14-Eicosadienoic acid, (Z)	polyunsaturated long-chain fatty acid	3.16	Nematicide, pesticide, antimicrobial, anti-inflammatory anti-androgenic flavour, and anticancer.	^27^
19.3621	Thymol-.beta.-d-glucopyranoside, tetrakis(O-trimethylsilyl)-	Organosilicon	3.7149	Immunosuppressive, antiparasitic, pesticidal, and antimicrobial activities	^28^
19.6957	Sucrose	Disaccharide	6.71	Nonfood uses.	^29^
20.0081	1-Monolinolein, 2TMS derivative	Monoglyceride	1.80	Enhanced liver FFAs –oxidation	^30^
23.7625	2-(2-Bromo-4-methylphenoxy)-N’-([1-(4-nitrophenyl)−2-pyrrolidinyl]methylene)acethydrazide	Phenolic	6.41	Has significant antibacterial activity against *Myco tuberculosis*.	^31^

The results in Table 4 represent the GC-MS analysis of jatropha, *J. curcas* seeds cake, revealing the detection of 25 compounds.

**Table 4:** Major bioactive compounds extracted from jatropha seed cake, *Jatropha curcas*, using GC-MS analysis.

Rt	Compounds	Area%	Classification	Activity	Ref.
8.2897	Glycerol	6.31	Sugar alcohol	Used pharmaceutical industries	^22^
9.4441	1-(1-Butoxy-2-propoxy)−2-propanol	3.325	Organic	Used as mutagenic in-vitro	^32^
10.8474	D-Psicofuranose, pentakis (trimethylsilyl) ether (isomer 2)	3.8129	Organosilicon	Antioxidant and antimicrobial	^33^
11.2869	D-Glucitol,	2.48	Sugar alcohol	Used as a sweetener due to is less cariogenic than sucrose.	^34^
12.0441	Palmitic Acid	15.2	Saturated fatty acid	Antitumor	^23^
12.2665	9,12-Octadecadienoic acid	1.56	Unsaturated fatty acid	Antioxidant, anticancer, antimicrobial, anti-inflammatory, and hepatoprotective effects.	^35^
12.706	Oleic Acid	12.51	Unsaturated fatty acid	Cosmetic products	^36^
12.8384	Linoelaidic acid, trimethylsilyl ester	1.05	Unsaturated Fatty acids	Antioxidant	^37^
12.9973	2-((2-Methyl-1-oxa-4-azaspiro[4.4]non-4-yl)carbonyl)cyclopropanecarboxylic acid tms peak 2	1.18	Organic heterocyclic compound	Herbicidal, antibiotic, antitumour, antidopamine and antiviral activities	^38^
13.0396	9,12-Octadecadienoic acid (Z, Z)	1.22	Unsaturated Fatty acids	chloride Antisecretory Antispermigenic, Antitonsilitic, Antitubercular, Choleretic, Contraceptive	^24^
13.1244	Eicosanoic acid	2.1684	Unsaturated Fatty acids	Antidiabetic, Hypolipidemic and antioxidant	^39^
13.1985	[4-Bromo-2-(hydrazono-phenyl-methyl)-phenyl]-carbamic acid, ethyl ester	1.24	Phenol	Used as a treatment for Alzheimer’s disease	^40^
13.3944	benzenesulfonamide, 4-[2-(4-hydroxy-6-methoxy-1-naphthalenyl)diazenyl]	1.06	Phenol	Antioxidant	^41^
13.4897	1,2-Benzenedicarboxylic acid, dipropyl ester	1.89	Organic acid	Antimicrobial	^42^
13.5798	1-Monopalmitin	1.79	Glycerides	Used in food, cosmetic, and pharmaceutical industries	^43^ ^44^
13.7227	[4-Bromo-2-(hydrazono-phenyl-methyl)-phenyl]-carbamic acid, ethyl ester	1.11	Phenol	Used as a treatment for Alzheimer’s disease	^40^
13.9875	Sucrose,	4.01	Disaccharide	Nonfood uses.	^29^
14.0405	2-linoleoylglycerol	1.23	Unsaturated fatty acids	Antibacterial and antifungal activity	^45^
14.2099	1-Monolinolein,	2.48	Glycerides	Antioxidant	^46^
14.4482	Maltose	2.95	Disaccharide	Consumed on a very regular basis in the human diet	^47^
15.5602	3-Trimethylsilyloxy-5-nonyne	2.08	Organosilicon	Anti-diabetic Activity	^48^
16.3598	1 H-Indene, 2,3-dihydro-1-methyl-3-octyl	2.21	Hydrocarbon	Recycling of the water-phase from hydrothermal conversion of biomass	^49^
16.4922	Indan, 1-methyl-3-nonyl	1.96	Hydrocarbon	Antimicrobial and antioxidant Activity	^50^
16.974	trans-4-Aminocyclohexanol, trimethylsilyl ether	1.68	Organic	Antioxidant	^51^
17.1276	1 H-Indene, 2,3-dihydro-4-propyl	3.46	Hydrocarbon	Antimicrobial, Anticancer, nematicide, hepatoprotective, Anti-arthritic, anti-asthma, diuretic	^52^

The results in Table 5 demonstrate the GC-MS analysis of jojoba, *S. chinesis* seed cake, highlighting the presence of several chemicals identified by GC-MS analysis. These components are Trifluoroacetamide (11.8373%); Palmitic Acid (3.0529%); 9-Octadecenoic acid (5.5698%); (E),-Octadecen-1-ol (2.9755%); Di-n-octyl phthalate (3.7327%); 13-Docosenoic acid, (Z)-(2.0938%); Sucrose (2.7626%); 2-(2-Bromo-4-methylphenoxy)-N’-([1-(4-nitrophenyl)−2-pyrrolidinyl] methylene) acethydrazide (2.1054%); Methyl galactoside (1 S,2 S,3 S,4R,5R)-(4.7299%); 2-(2Bromo 47-Trimethylsilyloxy-7-methyloctanoic acid, trimethylsilyl ester (3.6636%); -methylphenoxy)-N’-([1-(4-nitrophenyl) pyrrolidinyl] methylene) acethydrazide (3.1533%); Alpha-D-Xylopyranose (2.8073%); D-Arabinose (2.7905); Diboroxane, triethyl[(4-methyl-2-pyridyl)amino] (2.2095%). However, certain compounds, namely Trifluoroacetamide; D-(-)-Tagatofuranose, pentakis(trimethylsilyl) ether (isomer 2); Indan, 1-methyl-3-nonyl; 1 H-Pyrrole-2,5-dicarbonitrile and 7-Trimethylsilyloxy-7-methyloctanoic acid, trimethylsilyl ester, exhibit antimicrobial and antifungal properties and have been confirmed for bio-pesticide activity.

**Table 5:** Major bioactive compounds extracted from Jojoba seed cake, *S. chinesis*, using GC-MS analysis.

Rt	Compounds	Area%	Classification	Activity	Ref.
6.2–7	Trifluoroacetamide	11.83	Organic	Control of agricultural pests, Antifreezing agents, or de-icing products	^54^
10.042	Silanol, trimethyl-, phosphate (3:1)	1.52	Organosilicon	Antioxidant activity	^55^
12.171	1-(1-Butoxy-2-propoxy)−2-propanol	1.08	Alcohol	Highly effective herbicides.	^56^
12.224	Tripropylene glycol mono-n-butyl ether, TMS derivative	1.22	Organic	As a draw solution	^57^
14.591	D-(-)-Tagatofuranose, pentakis(trimethylsilyl) ether (isomer 2)	1.04	Organosilicon	Fungicide used in food preservative	^58^
15.793	Palmitic Acid	3.05	Saturated fatty acids	Antitumor activity in mice.	^23^
*16.8309*	9-Octadecenoic acid, (E)	5.56	Unsaturated omega-9 fatty	Hydroxy fatty acids, Growth inhibition of plant pathogenic fungi	^59^
16.9951	Stearic acid, TMS derivative	1.47	Saturated fatty acid	Microbial production	^60^
17.4769	-Octadecen-1-ol, (Z)	2.97	Unsaturated alcohol	Sex pheromone of the rice leaf folder.	^61^
17.9641	13-Eicosenoic acid, (Z)-,	5.69	Omega-7 Unsaturated fatty acid	Biopesticides for agricultural applications	^61^
18.5572	9-Octadecen-1-ol, (Z)	3.4584	Unsaturated fatty acid alcohol	Biological activity	^62^
18.7425	Di-n-octyl phthalate	3.73	Organic	Synthetic chemical esters impart flexibility, pliability and elasticity to plastics	^63^
18.7955	Diisooctyl phthalate	1.12	Organic	Public health concerns that it is carcinogenic, teratogenic, hepatotoxic and endocrine effects.	^64^
19.0179	13-Docosenoic acid (Z)	2.09	Unsaturated fatty acid	Major constituents in the oil of medicinal plants that uses as excellent antibacterial activity	^48^
19.0814	[4-Bromo-2-(hydrazono-phenyl-methyl)-phenyl]-carbamic acid, ethyl ester	1.31	Organic acid	Have biological activities, thus may be useful in traditional medicine and as a health supplement	^65^
19.3886	Sucrose	2.76	Disaccharide	Nonfood uses.	^29^
19.5209	7-Tetradecen-1-ol, (Z)	1.92	Unsaturated fatty acid alcohol	Sex pheromone component of the spotted cutworm	^66^
19.7487	2-(2-Bromo-4-methylphenoxy)-N’-([1-(4-nitrophenyl)−2-pyrrolidinyl]methylene)acethydrazide	2.10	Phenolic	controlling endoparasites and ectoparasites of warm-blooded animals	^67^
20.0028	Methyl galactoside (1 S,2 S,3 S,4R,5R),	4.72	Monosaccharides	Antioxidants and could inhibit tumor necrosis	^59^
20.167	2-(2-Bromo-4-methylphenoxy)-N’-([1-(4-nitrophenyl)−2-pyrrolidinyl]methylene)acethydrazide	3.15	Phenolic	Has significant activity against *Myco tuberculosis*.	^68^
20.31	Methyl.beta.-Arabinofuranoside	1.72	Complex polysaccharides	Initiation of chromosome replication	^69^
20.3841	Alpha.-D-Xylopyranose,	2.80	Complex monosaccharides	Strong inhibitor activity. Used in enzyme analysis. Pentose and glucoronate interconversion.	^70^
20.47	D-Arabinose	2.79	Monosaccharides	Anticancer and antiviral	^71^
20.54	D-Lyxose	1.13	Monosaccharides	Used in food and pharmaceutical industries	^72^
21.71	Diboroxane, triethyl[(4-methyl-2-pyridyl) amino]	2.20	Flavonoid	Use in reduction of CO_2_ to CO	^73^
21.84	Urs-12-en-28-al, 3-(acetyloxy)-, (3.beta.)	1.94	Terpenoids	Enhance photodynamic activity in methoxyethyl substituents	^74^
22.09	Indan, 1-methyl-3-nonyl	1.78	Hydrocarbon	Anti-microbial	^75^
22.63	1 H-Pyrrole-2,5-dicarbonitrile	1.35	Alkaloid	Antimicrobial, antibacterial, antifungal, antiallergic, antiinflammatory, anticancer, and anticonvulsant activities	^76^
23.42	Beta.-Sitosterol,	1.29	Organic	Use on chronic ailments such as cardiovascular diseases and diabetes	^77^
23.88	7-Trimethylsilyloxy-7-methyloctanoic acid, trimethylsilyl ester	3.6636	Organosilicon	Antimicrobial	^27^
24.70	D-(+)-Glucosamine	1.97	Amino sugar	Enhanced degradation of a-chitin materials. Synthesis of several antibiotics and anti-carcinogens.	^78^^79^,

The GC-MS analysis shown in Table 6 identified 25 compounds in olive, *O. europaea* seed cake. However, the major detected compounds exhibited various biological activities.

**Table 6:** Major bioactive compounds extracted from olive, *O. europaea*, using GCMS analysis

Rt	Compounds	Clasification	Area %	Activity	Ref.
6.5846	Trifluoroacetamide	Organic	2.26	Control of agricultural/horticultural pests	^54^
10.0424	Glycerol	Sugar alcohol	5.32	Used in pharmaceutical and agrochemical industries	^22^
12.4677	Tyrosol	Phenethyl alcohol	2.16	Antioxidants, antiviral, anti-inflammatory, and antibacterial agents	^80^
13.7015	L-(-)-Arabitol, 5TMS derivative	Sugar alcohol	1.58	Useful and economic biorefinery building block.	^81^
13.7545	Xylitol,	Sugar alcohol	2.16	food and pharmaceutical industries	^82^
14.1834	Tripropylene glycol monomethyl ether	Organic	5.39	Fuel additive, used to reduce dust in diesel engines.	^83^
14.4905	D-(-)-Fructofuranose, pentakis(trimethylsilyl) ether	Organosilicon	7.09	Antimicrobial activity	^84^
14.8029	Quininic acid	Organic acid	2.04	Used in the production of cosmetics and lubricants	^85^
14.9936	Beta.-D-Allopyranose	Monosaccharide	4.53	Anticancer	^86^^87^,
15.0359	Alpha.-D-(-)-Lyxopyranose,	Complex monosaccharides	1.23	Used in preparation of fucosidase, inhibitors.	^88^
15.2213	D-Sorbitol	Sugar alcohol	2.07	Used as drug, food, and cosmetic	^89^
15.306	D-Mannitol,	Sugar alcohol	2.09	As a valuable nutritive sweetener	^90^
15.3483	d-Galactose, 2,3,4,5,6-pentakis-O-(trimethylsilyl)-, o-methyloxyme, (1Z)	Organosilicon	1.03	As cytotoxic, immunosuppressive, pesticidal, anti-parasitic and antimicrobial activities	^29^
15.5443	D-Galactose,	Monosaccharides	2.36	Treatment to cause neuronal injury and reduced neurogenesis	^91^
15.7561	D-Gluconic acid	Organic acid	1.77	Cleaning agents,	^92^
15.8249	Palmitic Acid	Saturated fatty acids	3.70	Induces apoptosis in the human leukemic cell Palmitic acid also shows in vivo antitumor activity in mice	^23^
16.8575	9-Octadecenoic acid	Unsaturated omega-9 fatty	6.89	Antimicrobial oil/water separation	^93^
17.0217	Stearic acid	Saturated fatty acid	1.12	Use as the plasticizer in corn zein protein	^26^
19.7117	1-Monooleoylglycerol	Alcholic fatty acid	1.25	Determination of glycerin in biodiesel	^94^
19.8546	D-(+)-Trehalose, octakis(trimethylsilyl) ether	Organosilicon	1.07	Pharmaceutical and cosmetic sectors	^95^
19.8917	2-O-(2-(4-hydroxyphenyl)-ethyl)-d-.beta.-glucopyranose	Phenolic	1.63	Pesticidal, antimicrobial and anti-parasitic activities	^28^
19.9923	Maltose	Disaccharide	2.0	Human diet	^47^
20.1988	Methyl galactoside,	Flavonoid O-glycoside	1.78	Use as a chemotaxonomic marker	^96^
20.3312	Glyceryl-glycoside	Complex monosaccharides	1.20	In skin health	^97^
20.4477	3-.alpha.-Mannobiose, octakis(trimethylsily) ether, methyloxime (isomer 2)	Organosilicon	1.73	Antimicrobial, antioxidant, anti-cholinesterase	^98^

**Fig. 5:**
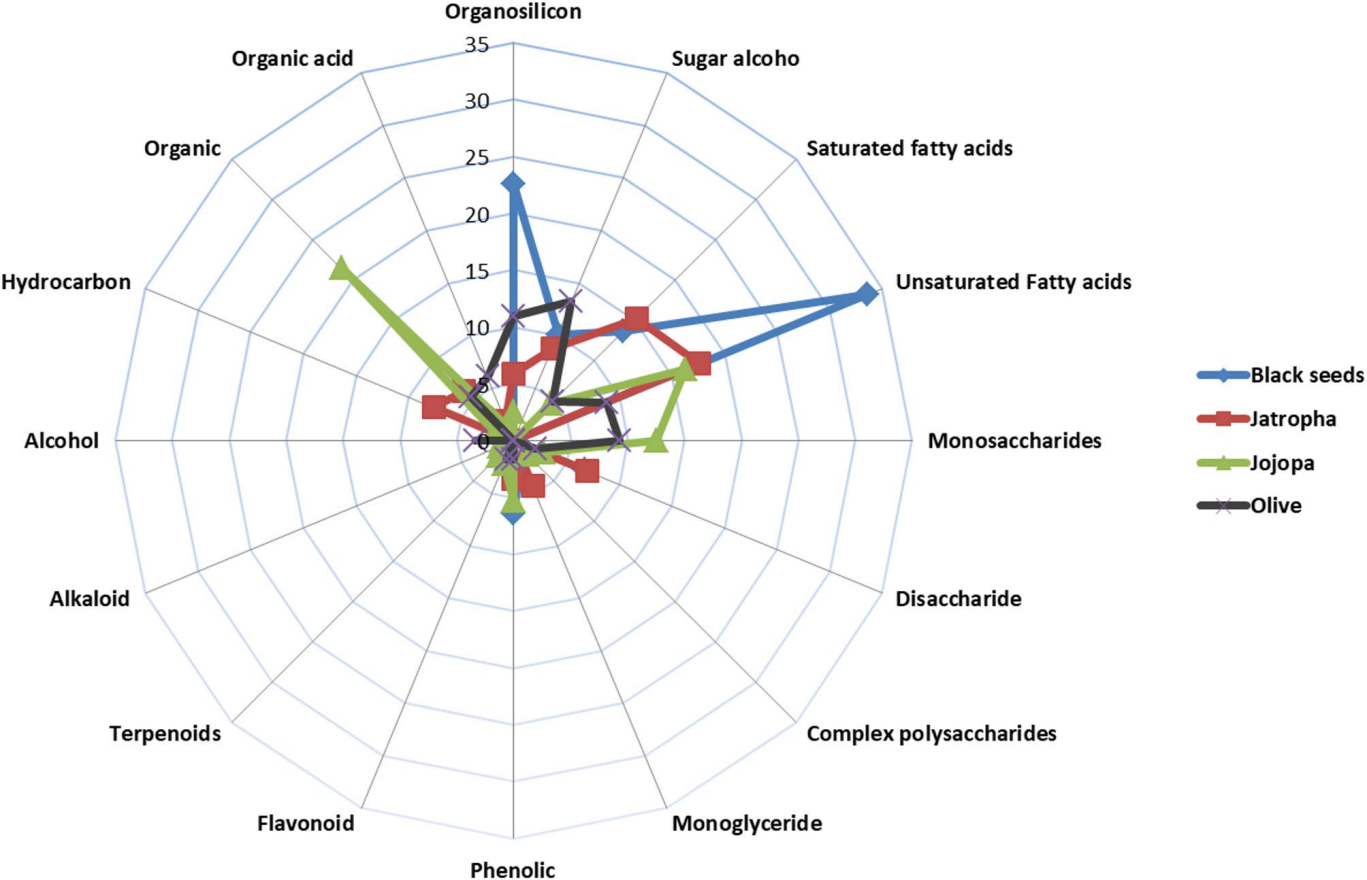
Radar analysis of the common constituents found in plant seed cakes.

Figure 5 demonstrates the most common constituents of plant seed cakes, including black seed, *N. Sativa*, jojoba, *S. chinesis*, olive, *O. europaea*, and jatropha, *J. curcas*. However, the most common constituents found in black seeds are unsaturated fatty acids and organosilicon compounds. The most prevalent components in jojoba are monosaccharides and organic compounds. Sugar alcohols are the predominant compounds found in olive seed cake. Saturated fatty acids, hydrocarbons, disaccharide, and monoglycerides are the most active compounds in jatropha. The most prevalent ingredients found in black seed are unsaturated fatty acids, organosilicon, and phenolic compounds. Among the tested seed cakes, black seed exhibited the strongest impact against nematodes.

Finally, the results indicated the active compounds present in the extracts obtained through GC-MS analysis of four seed cakes. For example, one of the compounds found in black seeds at a high percentage is Bis(trimethylsilyl) trifluoroacetamide 18%, which possesses anti-inflammatory, antibacterial, antiulcerative and antitumor properties^21^. Palmitic acid (10.6921%) induces apoptosis in human leukemic cells and exhibits antitumor activity^23^9,12-Octadecadienoic acid (Z, Z)-(13.6856%) has antisecretory, antispermigenic, antitonsilitic, and antitubercular properties^24^Linoelaidic acid, trimethylsilyl ester (16.7845%), which has antioxidant and anti-inflammatory properties also acts as an anticancer agent, 2-(2-Bromo-4-methylphenoxy)-N’-([1-(4-nitrophenyl)−2-pyrrolidinyl]methylene)acethydrazide (6.4194%) exhibits significant antibacterial activity against *Tuberculosis*^31^. According to GC-MS analysis, the major component found in jojoba seeds was trifluoroacetamide (11.83%), which can be used to control agricultural and horticultural pests, as well as in antifreezing agents, or deicing products^54^followed by minor derivatives, such as methyl galactoside (5.5698%), which is used as an inhibitor of plant-pathogenic fungi^59^and 13-Eicosenoic acid, (Z)-(5.6966%) which acts as a bio-pesticide for agricultural applications^61^. The most active compounds found in olive seeds include D-(-)-Fructofuranose, pentakis (trimethylsilyl) ether, which has antimicrobial activity^83^Beta-D-Allopyranose, which inhibits the gene expression and secretion of pro-inflammatory cytokines in a dose-dependent manner^86^and 9-Octadecenoic acid (6.89%), which exhibits antimicrobial activity^92^. Jatropha cake exhibited anti-nematode activities due to its major constituents, including palmitic acid (15%), which has antitumor activity in mice^23^and oleic acid, (12.51%), which is used in cosmetic products^36^controls agricultural and horticultural pests^54^and exhibits a wide variety of biological properties, such as pesticidal, anti-parasitic and antimicrobial activities^28^. These reports reveal the presence of plant bioactive components isolated in the methanol extract of the current plant seed cakes.

Thus, our research could be useful in the development of new natural control methods against pests, such as RKNs (*M. incognita)* infested pepper (*Capsicum* spp.*)*. Consequently, this approach is a valuable tool for systematically enhancing plant health. Natural fatty acids can effectively control parasitic nematodes without causing environmental pollution^90^. Interestingly, these bioactive substances extracted from seeds exhibit nematicidal characteristics, potentially through various mechanisms. Certain substances interfere with nematode neurotransmission, leading to paralysis and death^105^. Bioactive compounds affect nematode development, egg-laying, and J2s motility, indicating a disruption of the cuticle and other physiological processes^106^. Silicon compounds stimulate plant resistance against nematodes by enhancing physical barriers and activating defense-related enzymes^107^. Unsaturated fatty acids contain bioactive chemical substances with a diverse range of biological activities^98^. Phenolic compounds could be considered strong nematocidal agents and antioxidants, protecting plants from diseases and integrating into pest-control systems^99,100^.

## Materials and methods

### Preparation of RKNs, *M. incognita* in pure culture

Eggs of RKNs, *M. incognita*, were obtained from the roots of tomato plants (*Lycopersicon esculentum* cv. Castle Rock) infected with the RKNs using a (0.5% NaOCl) solution^13^. Juveniles2 (J2s) were collected daily from pure egg masses and were stored at 15 °C. The J2s larvae used in the study were less than five days old.

## Nematode management experiments

### Cakes

Black seed, *Nigella sativa*, Jojoba, *Simmondsia chinensis*, Olive, *Olea europaea* and Jatropha, *Jatropha curcas* cakes were purchased from Haraz Company in Cairo, Egypt, with permission, in accordance with institutional, national, and international guidelines and legislation.

### Treatments

The treatments of the seeds cake are used as follows: (A) *N. sativa* 15 g, (B) *N. sativa* 30 g, (C) *S. chinensis* 15 g, (D) *S. chinensis* 30 g, (E) *O. europaea* 15 g, (F) *O. europaea* 30 g, (G) *J. curcas* 15 g, (H) *J. curcas* 30 g, I), Nematicides check, Oxamyl 0.5 ml (Vydate 24% L {Vydate L24%, Methyl-N, N-dimethyl N- (Methyl carbamayl) oxythioxamidate}); J), Check with infection; K), Check without infection.

### In vivo trial

The current study was conducted in the greenhouse of the Molecular Nematode Diseases Laboratory, Department of Plant Biotechnology, Genetic Engineering and Biotechnology Research Institute (GEBRI), University of Sadat City (USC), Menofia Governorate, Egypt. All tested treatments were applied to pepper (*C. annum* L.) plantlets grown in 30 cm pots filled with steam-sterilized sandy and clay soil (3:1, V: V). Fifty pots were infested with 3,000 active J2s, *M. incognita* per pot at the time of seedling transplantation. Seven days later, 40 pots were treated with the previously mentioned materials (each treatment replicated five times) after preparation. Also, five of the infested pots were treated with 0.5 mL of oxamyl per pot (chemical control). Likewise, five pots remained without nematode inoculation (check). In addition, five pots were inoculated at the previously mentioned level (nematode check). All pots were arranged in a row design on a clean bench in the greenhouse and placed in a completely randomized design. After two months, the trial ended, and the plants were harvested. The roots were washed carefully with water to remove fine soil particles and then stained with a synthetic solution of 3.5 g “Phloxine B” in 750 mL distilled water + 250 mL acetic acid (5% solution) for 5 min., to facilitate nematode counting in the root. Nematode variables were calculated per root, including galls, juveniles3 (J3), juveniles4 (J4), females, and egg-laying females. Larval J2s per 250 g of soil were extracted and counted using Cobb’s sieving and decanting method^101^with sieves (20, 60 and 325 meshes). Plant growth parameters, including shoot length and weight, root length and weight, and the number of leaves, were recorded.

### Methanol extracts

Approximately 10 g of each plant material (jatropha, black seed, jojoba, and olive cakes) was separately immersed in 200 mL of methanol (1:20 w/v) and stirred for 1 h. After that, the mixtures were filtered, and the filtrates were collected and evaporated.

### GC-MS analysis of methanol extract of the plant seed cakes

Chromatographic analysis using GC-MS was conducted at the Chromatography Laboratory Analyst, GC-MS, IC Central Lab., Faculty of Science, Ain Shams University. The analysis was performed using an Agilent Technologies 7890B GC System combined with 5977 A Mass Selective Detector. A capillary column (HP-5MS Capillary) was used with helium as the carrier gas, and a 1 µL injection was made. The constituents were identified based on mass fragmentation with the NIST/EPA/NIH Mass Spectral Library (NIST14) and NIST Mass Spectral Search Program (Version 2.2).

### Statistical analysis

Each experiment was conducted in five replicates, and the data values were expressed as mean ± SEM. All data were subjected to analysis of variance (ANOVA)^14^. The significance of the mean differences was determined using Duncan’s multiple range tests comparing the treatments against the control group. Data analyses were performed using SPSS 16 software (*p* ≤ 0.05).

## Conclusions

Root-knot nematodes (RKNs), *Melidogyne incognita* pose a significant challenge for farmers worldwide, leading to substantial yield losses. Various conventional strategies, including synthetic nematocides (toxic materials), have been used in the past to manage plant-parasitic nematodes (PPNs) in crops and soils. Therefore, in a greenhouse experiment aimed at reducing RKN, *M. incognita* populations in soil and root infestation, we investigated the effectiveness of plant seed cakes as natural control agents. Recently, the efficacy of four plant seeds cakes (PSCs) in managing PPNs has been investigated, including *Nigella Sativa*, *Simmondsia chinesis*, *Olea europaea*, and *Jatroph curcas.* These seed cakes inhibited nematode reproduction and enhanced plant health. Notably, *N. Sativa* and *S. chinesis* exhibited the strongest nematicidal effects against *M. incognita* juveniles (J2s) in soil. Treatment with black seed at both 15 and 30 g. rate as well as jojoba at 15 g, was highly effective in decreasing gall count (188, 107.9 and 179.5, respectively). These treatments also significantly decreased the numbers of J3, J4, females and egg masses in the plant, as well as in J2s in the soil, and the final nematode population and the rate of build-up. Data on growth parameters, including the weight and length of shoots and roots, as well as the number of leaves, were measured. The results showed that black seeds at 30 g, and jojoba at 15 g led to a significant increase in shoot weight, followed by black seeds at 15 g (75.89, 47.86 and 45.9 g, respectively). Overall, the seed cake extracts of the tested treatments in this research demonstrated a strong antagonistic effect against RKNs. However, they may contain natural nematotoxic compounds that can suppress or eliminate the RKNs in soil and roots. Some of these PSCs contained amines, indoles, tannins, and saponins. All the tested treatments demonstrated toxicity against RKNs. These PSCs, for instance, *N. sativa*,* S. chinensis*, *O. europaea*, and *J. curcas* may be used as bio-pesticides, especially as anti-nematodal agents that reduce nematode populations and enhance plant health. Based on the obtained results, the use of natural materials for controlling RKNs is highly recommended. These compounds should be administered in prescribed doses, taking into account the crop type, pest characteristics, and environmental conditions. Further research on bioactive components is needed to evaluate their efficacy through in vitro and in vivo studies and to confirm their effectiveness and safety in open-field applications for the treatment of various plant diseases.

## Data Availability

The datasets spent and/or analyzed during this study are available from the corresponding author upon reasonable request.
